# A novel wastewater-based epidemiology indexing method predicts SARS-CoV-2 disease prevalence across treatment facilities in metropolitan and regional populations

**DOI:** 10.1038/s41598-021-00853-y

**Published:** 2021-11-01

**Authors:** Richard G. Melvin, Emily N. Hendrickson, Nabiha Chaudhry, Onimitein Georgewill, Rebecca Freese, Timothy W. Schacker, Glenn E. Simmons

**Affiliations:** 1 Department of Biomedical Sciences, University of Minnesota Medical School, Duluth, MN USA; 2 National Summer Undergraduate Research Program, University of Maryland, College Park, MD USA; 3 Biostatistical Design and Analysis Center, Clinical and Translational Science Institute, University of Minnesota, Minneapolis, MN USA; 4 Department of Medicine, University of Minnesota Medical School, Minneapolis, MN USA; 5 Carcinogenesis and Chemoprevention Program, Masonic Cancer Center, Minneapolis, MN USA

**Keywords:** Biological techniques, Epidemiology

## Abstract

There is a need for wastewater based epidemiological (WBE) methods that integrate multiple, variously sized surveillance sites across geographic areas. We developed a novel indexing method, Melvin’s Index, that provides a normalized and standardized metric of wastewater pathogen load for qPCR assays that is resilient to surveillance site variation. To demonstrate the utility of Melvin’s Index, we used qRT-PCR to measure SARS-CoV-2 genomic RNA levels in influent wastewater from 19 municipal wastewater treatment facilities (WWTF’s) of varying sizes and served populations across the state of Minnesota during the Summer of 2020. SARS-CoV-2 RNA was detected at each WWTF during the 20-week sampling period at a mean concentration of 8.5 × 10^4^ genome copies/L (range 3.2 × 10^2^–1.2 × 10^9^ genome copies/L). Lag analysis of trends in Melvin’s Index values and clinical COVID-19 cases showed that increases in indexed wastewater SARS-CoV-2 levels precede new clinical cases by 15–17 days at the statewide level and by up to 25 days at the regional/county level. Melvin’s Index is a reliable WBE method and can be applied to both WWTFs that serve a wide range of population sizes and to large regions that are served by multiple WWTFs.

## Introduction

Wastewater based epidemiology (WBE) is well established as a method for surveying the prevalence of pathogens in a community or regional population. During disease outbreaks, WBE can be used to identify localities that are at high risk for disease spread and to guide the deployment of public health interventions before clinical cases increase. WBE is also an established strategy for the monitoring and control of pathogens in large populations^1^. Limitations of current WBE monitoring studies are the almost exclusive focus on major population centers (≥ 100,000 persons), the effects of system variation between wastewater treatment facilities (WWTFs) on determining pathogen load, and inconsistent normalization of reporting metrics^2,3^. In this study we monitored the presence of SARS-CoV-2 genomic RNA levels in influent wastewater from 19 municipal WWTFs of varying sizes and served populations across the state of Minnesota during summer 2020. The goal of this study was to address these issues through inclusion of WWTFs that service populations of varying sizes, by accounting for WWTF system factors that influence the concentration of pathogens in wastewater (e.g., population size, flow rate), and by developing an indexing method, Melvin’s Index, for normalizing and standardizing the results of qPCR tests.

**Figure 1:**
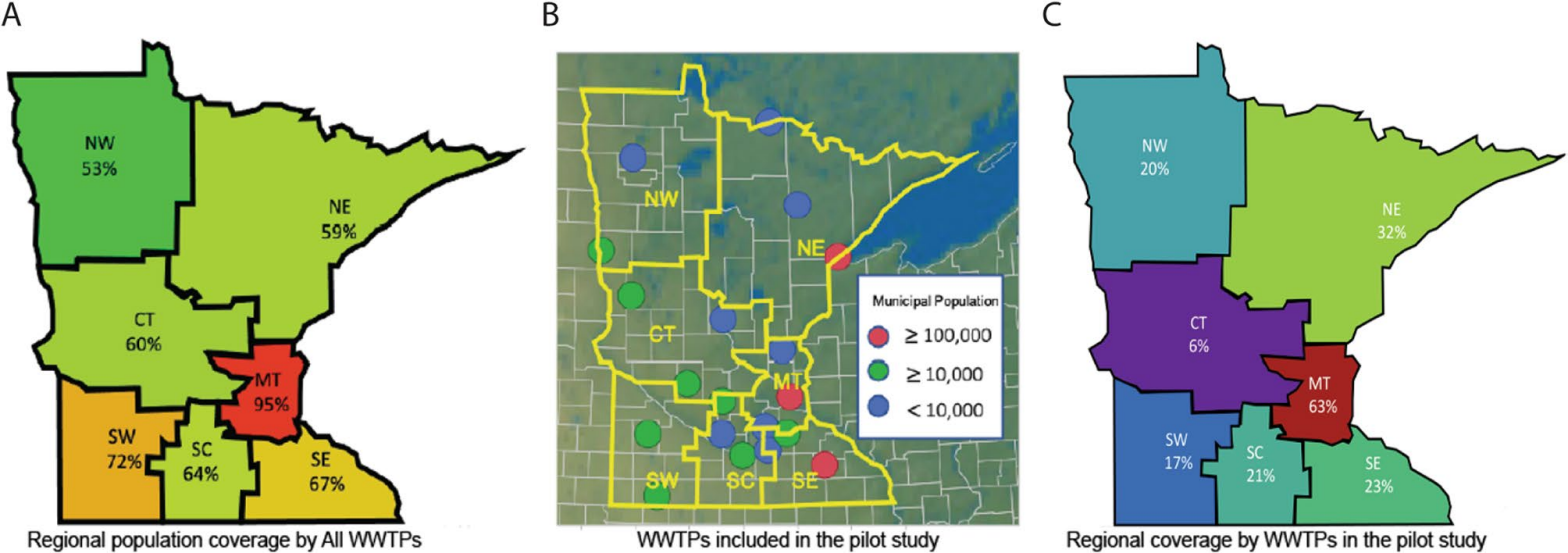
Population coverage of the wastewater surveillance study. (**A**) Estimated percentage of the population in each region of Minnesota that is served by a municipal wastewater treatment facility. (**B**) Location of the 19 wastewater treatment facilities included in the study. (**C**) Estimated percentage of the regional population represented in the study.

To determine how well wastewater surveillance performs across a range of population levels, this study monitored large (> 100,000 population), medium (100,000 to 10,000), and small (< 10,000) communities in Minnesota. Minnesota is the 12th largest U.S. state by area (225,163 km^2^) and 22nd largest by population (5.64 million, the median population of a U.S.A. state is 4.47 million). More than half of the population of Minnesota (58.7 percent) resides in the Minneapolis/St. Paul metropolitan region and the remaining 41.3 percent resides in six geographic regions outside of the metropolitan area (Fig. 1A,B). Based on the population served by WWTFs, there is the potential to monitor 81.4% of the Minnesota. Active WWTFs could provide surveillance coverage of 95% of the metropolitan population and from 53 to 72% coverage in regional Minnesota (Fig. 1A). For this study we recruited 19 WWTFs that ranged in service population size from 500 to 2 million served population and covered 42.2% of the state population (Fig. 1B,C, Table S1).

One problem of monitoring large geographical regions is that WWTF systems vary greatly in served population and influent wastewater flow rate. Variation in these system parameters can have a diluting or concentrating effect that can influence the detection of pathogens. To compensate for these effects influent flow rate is commonly included in the calculations of pathogen concentration. In this study we analyzed the effects of served population size, influent wastewater flow rate, and their interaction on detection of a plant virus that is commonly found in human waste, the pepper mild mottle virus (PMMoV)^19^. Influent flow rate will influence pathogen concentration that parallels that of PMMoV and may influence detection. Because of this effect, an analysis method is required that eliminates the influence of flow rate. A common approach to remove the effect of flow rate is to standardize pathogen concentrations to that of PMMoV.

In this study we developed a normalized and standardized WBE index, Melvin’s Index, that can be used for benchmarking and tracking pathogens in wastewater. Pathogen surveillance by WBE is conducted by sampling influent wastewater and then assaying for the presence of a molecular signature of the pathogen of interest. The molecular signal is commonly a targeted gene within the pathogen genome that can be detected and quantified using pathogen specific primer/probes in a quantitative polymerase chain reaction (qPCR). For example, SARS-CoV-2, the virus that causes the disease COVID-19 is detected and quantified by qPCR after reverse transcribing the viral genome. The results of qPCR testing are often reported as the reaction cycle at which a detectable signal of the target molecule is attained (cycle threshold, C_T,_ or quantification cycle, C_q_). When C_T_ or C_q_ is compared to a standard curve, the results are often interpreted and reported as the logarithm of pathogen genomes per unit volume. The meaning of a C_T_-value or of the log concentration of genomes can have meaning when tracked over time and compared to previously determined values. However, if these values are not standardized then fluctuations in their levels may only reflect variation in WWTF parameters such as the flow rate of influent wastewater or in population size differences between WWTF’s. Moreover, qPCR primer/probe sets amplify and detect their targets with varying efficiencies over a range of concentrations so that C_T_-values may not be comparable between targets. Quantile normalization is a common first step for comparing qPCR results from different primer/probe sets^4^. The normalization procedure adjusts the overall detection level so that the data distribution for all primer/probe sets is equal and comparable. Melvin’s Index employs quantile normalization to equalize the data distributions of the pathogen specific and of an internal standard primer/probe. Normalized pathogen specific data are then standardized to the corresponding normalized internal standard.

Several studies have highlighted the utility of WBE for monitoring prevalence and incidence of COVID-19 in communities^5–8^. WBE of SARS-CoV-2 in large populations is informative as it is estimated that approximately 80 percent of COVID-19 cases are mild or asymptomatic^9^. Given that there may be many infected individuals that are asymptomatic or paucisymptomatic and not partaking in testing, WBE applied over time may be the most effective tool for assessing community disease prevalence and incidence. The data presented herein suggests that WBE surveillance provided early detection SARS-CoV-2 for both large and small communities. However, the power of WBE is largely enhanced when raw data are converted to a standardized and normalized index value like Melvin’s Index. We saw that applying the index to measured SARS-CoV-2 concentrations reduced the influence of WWTF system factors that alter the concentration of pathogens in wastewater.

## Results

### Monitoring SARS-CoV-2 in wastewater in the state of Minnesota

We successfully recruited fifteen regional and four metropolitan area WWTFs with at least one WWTF participating from each region of the state of Minnesota, USA. Of the nineteen communities serviced by WWTFs that participated in the study, there were three large, eight medium, and eight small communities (Table S1). We performed a longitudinal study of SARS-CoV-2 levels over 20 weeks (May to August 2020) 19 WWTFs and all WWTFs participated for the full study period. The proportion of the regional population that was monitored through participating WWTFs ranged from 6.3 to 63 percent (Table S1, Fig. 1B,C).

### Effect of WWTF system characteristics on detection of virus in influent wastewater

At the beginning of the study, we predicted that increases in the number of COVID-19 cases would increase the amount of SARS-CoV-2 RNA detected in that community’s wastewater. However, there are several factors that could affect the concentration of SARS-CoV-2 in the wastewater systems including daily flow rate, population size, and size of the WWTF (designed daily flow rate). To better illustrate the magnitude of the differences between each facility, we ranked facilities based on their daily flow rate, daily flow rate per capita, and designed flow rate per capita (Fig. S1A–C). As expected, larger WWTFs had larger daily flow rates (Fig. S1A). However, when facility water flow rates were ranked per capita, the rank order changes dramatically and reveals a concentrating effect on PMMoV at the largest site (Metropolitan, PMMoV cycle threshold (C_T_)-value of 25.2 ± 2.0, mean ± standard deviation, Fig. S1D), but a dilution effect at the second largest site (WLSSD, PMMoV C_T_-value of 28.8 ± 2.6, Fig. S1D). The concentration effect observed for the Metropolitan WWTF is likely due to the large population served by that facility. The Metropolitan WWTF serves 1.9 million while the median population served by WWTFs included in the study is 13,000. The dilution effect on PMMoV that was observed for WLSSD was likely due to flow from industrial wastewater over the sampling period (Fig. S1B). A similar, but less dramatic observation, was seen when ranking facilities based on designed waterflow capacity per capita (Fig. S1C).

### Tests for association of sewer system parameters on PMMoV standard C_T_-values

It was important to understand whether variation in system properties were associated with PMMoV levels because the global distribution of PMMoV C_T_ was used to calculate our SARS-CoV-2 index (Melvin’s Index). Use of the global distribution assumes that the level of PMMoV in wastewater is fairly constant within and between populations and that there is a general relationship between PMMoV level and flow rate of the sewer system^10–12^. SARS-CoV-2 level, on the other hand, was expected to vary greatly within and between populations and be subject to the same associations with flow as is PMMoV.

We observed that WWTFs could be ranked according to their mean PMMoV C_T_-values (Fig. S1D). This suggested a potential association with one or more site-specific variables. To better understand which site-specific variables influence the ranking of WWTFs by PMMoV C_T_, we tested whether flow rate, facility size (designed flow rate), population size, or the per capita measures of flow rate were associated with the ranking of WWTFs. When considering all the participating WWTFs together, per capita flow rate was significantly associated with ranked PMMoV C_T_-values (Kendall’s τ = 0.15, P < 0.01, Table S2, Fig. S1D). There was no significant association between PMMoV C_T_ ranking and flow rate, designed flow rate (facility size), size of the served population, or per capita measure of designed flow rate (Table S2).

Visual inspection of the per capita flow rate overlaid onto ranked PMMoV C_T_-values (Fig. S1D) suggested that their association may be due to the concentration and dilution effects previously noted for two of the larger WWTFs. Metropolitan and WLSSD have strikingly different per capita flow rates (Fig. S1B,D). The Metropolitan WWTF is a concentrated system that serves a population of 3,454,290 with a per capita flow rate of 0.55 ± 0.5 MGD/10 k population (mean ± standard deviation) and PMMoV C_T_ of 25.17 ± 2.00. WLSSD is a dilute system that serves a population of 137,590 with a per capita flow rate of 2.23 ± 0.20 MGD/10 k population (four times higher than that of Metropolitan) and PMMoV C_T_ of 28.44 ± 2.42. Significant association of the per capita flow rate with ranked PMMoV-C_T_ was lost when the WLSSD and Metropolitan WWTFs were removed from the analysis (Kendall’s τ = 0.08, P = 0.13), but not when either was removed individually (τ = 0.12, P < 0.05 and τ = 0.11, P < 0.05, respectively when Metropolitan or WLSSD were excluded) (Fig. S1E, Table S2). Although rankable, PMMoV C_T_, did not differ significantly between WWTFs. This finding demonstrated that even large local differences in flow rate have a small effect on PMMoV C_T_ and supported both the use of PMMoV as a standard and the global PMMoV distribution in the calculation of Melvin’s index.

After observing the wastewater influent flow rate characteristics for each facility, we sought to create a normalization and standardization strategy that would account for fluctuations in each system and allow for comparisons between WWTFs. This is an important step toward distinguishing “hot spots” in a large geographic area. To address system variation, we chose Pepper Mild Mottle Virus (PMMoV), an abundant plant RNA virus found in the human diet, as a standard, but we first needed to determine the impact each environmental variable had on PMMoV C_T_-values in wastewater samples.

**Figure 2:**
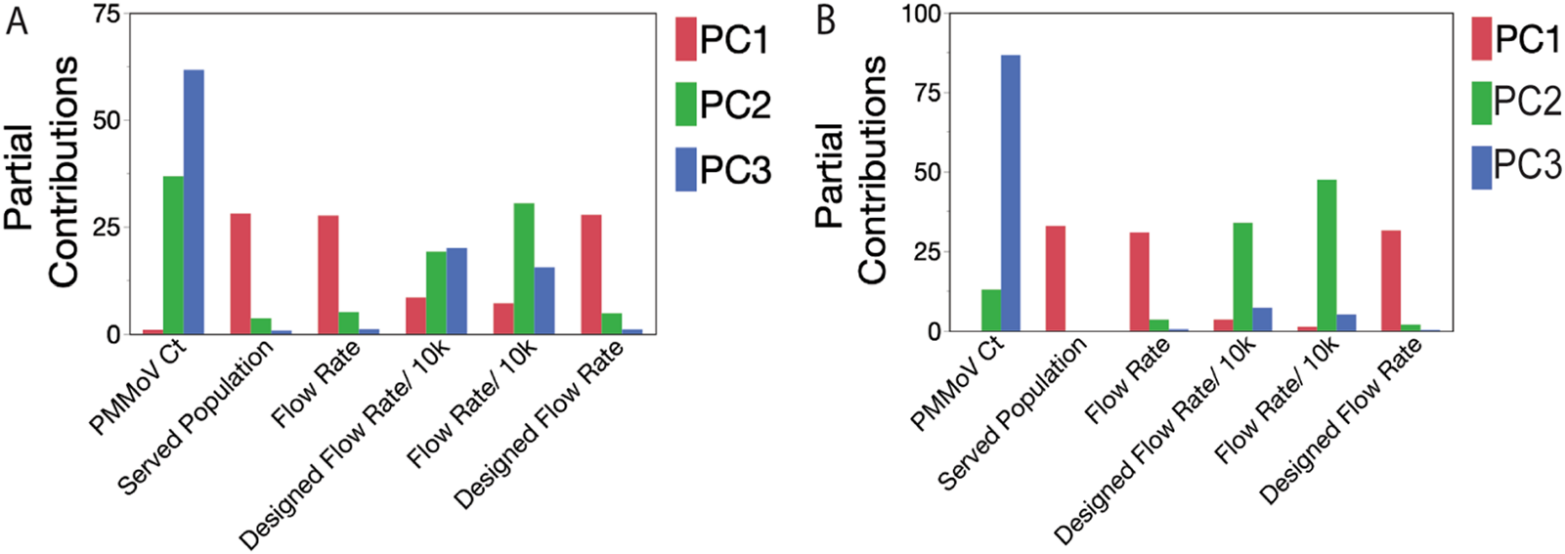
Determining the influence of flow rates and other variables on Pepper Mild Mottle Virus detection in municipal wastewater samples. (**A**) Principal component analysis of the effects of most influential component on variables in interest for all wastewater treatment facilities in the study. (**B**) Principal component analysis of the effects of most influential components on variables in interest for wastewater treatment facilities in the study, excluding of WLSSD and Metropolitan sites.

Tests of the relationship between wastewater influent flow rate variables and PMMoV C_T_-value showed that PMMoV C_T_-value was relatively stable across the study period and across WWTFs (Table S2). To further test for relationships between PMMoV C_T_-value, flow rate, served population size, and designed flow capacity of the WWTF, we performed a principal component analysis (PCA) on the correlation between variables. PCA combines variables that are associated with each other onto a common axis termed a principal component (PC) and quantifies the contribution of each variable to that axis. The strength of association between variables was inferred from their respective contributions to the PC axes that they share (Fig. 2A,B). PCA analysis that included all WWTFs showed that 74.2 percent of the variation between all variables was described by two principal components (PC1 and PC2) (Fig. 2A and Table S3). A further 15.3 percent of the variation was explained by PC3. PMMoV C_T_ contributed mainly to PC2 and PC3 (36.8 and 61.6 percent, respectively) with negligible contribution to PC1 (Fig. 2A). PCA showed a modest association between PMMoV C_T_, per capita flow rate, and per capita designed flow rate through their common contributions to PC2 (30.4 and 19.2 percent, respectively) and PC3 (15.4 and 20.0 percent, respectively) (Fig. 2A). Served population, flow rate, and designed flow rate made negligible contributions to PC2 and PC3 indicating very weak association with PMMoV C_T_.

To confirm that the modest association between PMMoV C_T_ and per capita flow rate was primarily due to the WLSSD and Metropolitan WWTFs, we removed both facilities from the analysis (Fig. 2B and Table S3). In agreement with the earlier tests, served population size, flow rate, and designed flow capacity contributed negligibly to PC3 (the axis that captured most of the PMMoV C_T_-value variation). Likewise, PMMoV C_T_ was a small contributor to PC2 (per capita flow) and a negligible contributor to PC1 (flow rate, designed flow rate, and population size). Taken together, these results showed that flow rate and population size had negligible to low-modest association with PMMoV C_T_ and confirmed our previous conclusions that PMMoV was a suitable standard for our index calculation.

### Melvin’s Index, a method for normalizing and standardizing qPCR assays of wastewater pathogen load

We created a normalized and standardized index of wastewater pathogen load, Melvin’s Index, to provide a simplified, scaled value for benchmarking and tracking pathogen levels. A goal of this study was to develop data analysis methods that are easily interpretable and could be applied to a wide range of population and WWTF sizes. Wastewater pathogen load has been reported as the C_T_-value obtained by quantitative polymerase chain reaction (qPCR) testing or as the logarithm of genome copies per liter of wastewater inferred by comparing standards to the qPCR C_T_-values^5,6,12–19^. While those measures hold meaning for experts, they can be opaque to the communities and officials who use the information. Additionally, data that are reported without appropriate normalization and standardization can make it difficult to discern the relative magnitude of disease prevalence in a population or between localities. For example, normalizing the number of new COVID-19 cases per capita showed that the disease was more prevalent in central Minnesota than in the metropolitan region in early May of 2020 (Fig. S2A).

Melvin’s Index addressed two issues of qPCR testing. The first issue was that of normalizing between different primer/probe sets. To address the issue, we applied quantile normalization (“Methods”, Fig. S2B,C). A second issue was that of samples which did not produce a reliably detectable amplification signal within the performed number of PCR cycles. Samples that do not amplify are often termed “negative tests”. Negative tests are either excluded or assigned a C_T_-value one cycle longer than the maximum cycles performed, which we refer to here as *C*_*T*_*-ceiling*. If *C*_*T*_*-ceiling* is not assigned appropriately then exclusion or inclusion of the data may create a bias that leads to misestimation of pathogen load.

In our study, to capture potentially low levels of SARS-CoV-2 genomic RNA in wastewater we extended PCR to 50 cycles. Because amplification at late cycles is infrequent, C_T_*-ceiling* of 51 may bias Melvin’s Index calculations. To reduce this potential bias, we calculated a C_T_ that bounded 95% of the data with coefficient of variation equal to 10% (CV10%) among measures using Log_10_ (genome copies/L)^20,21^. We then assigned *C*_*T*_*-ceiling*(*CV10%*) equal to one cycle greater than that of the 95% boundary C_T_ for each target. For N1 and N2, *C*_*T*_*-ceiling*(*CV10%*) = 43, and all raw C_T_-values greater than or equal to *C*_*T*_*-ceiling(CV10%)* were assigned the same value. For PMMoV, *C*_*T*_*-ceiling*(*CV10%*) = 33 and it was assigned in the same manner. To reduce the potential bias, we excluded the *C*_*T*_*-ceiling* data points from the quantile normalization step. However, following normalization, *C*_*T*_*-ceiling* data were added back to the first quantile of the normalized data. Details of the Melvin’s index calculation are provided in the Methods section. To test for bias in *C*_*T*_*-ceiling* assignment, we repeated analyses using *C*_*T*_*-ceiling(51)* = 51 for all targets and regressed against the calculated *C*_*T*_*-ceiling(CV10%)*. For N1 and N2, linear regression showed a linear relationship that indicated that use of *C*_*T*_*-ceiling(51)* would introduce minimal bias into the calculation of Melvin’s Index. The linear relationships were described by

$$ C_{T} - ceiling\left( {CV10\% } \right) = 0.0{2} + {1}.0{6} \times C_{T} - ceiling(51),\;{\text{R}}^{{2}} = 0.{91}\;{\text{for}}\;{\text{N1}} $$

and

$$ C_{T} - ceiling\left( {CV10\% } \right) = 0.00 \, + \, 0.{99} \times C_{T} - ceiling\left( {51} \right),\;{\text{R}}^{{2}} = 0.{94}\;{\text{for}}\;{\text{N2}}. $$

### Melvin’s Index values of SARS-CoV-2 levels from wastewater showed a similar trend to that of new clinical COVID-19 cases

**Figure 3:**
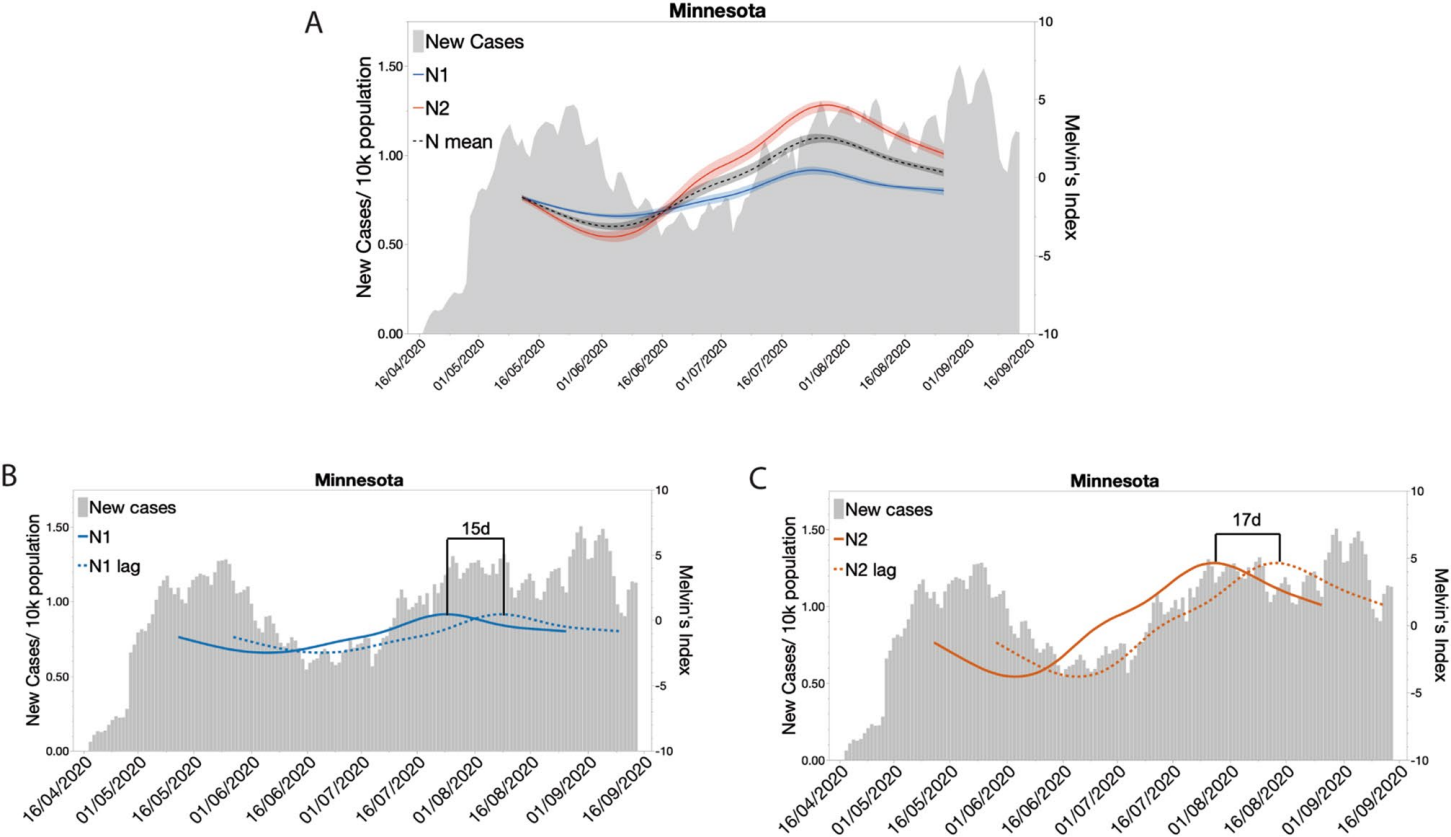
Indexed SARS-CoV-2 levels in wastewater trends across the state show a similar pattern to new cases per capita with a temporal offset. (**A**) New daily clinical cases of COVID-19 overlayed by the indexed SARS-CoV-2 N1, N2, and the mean of N1 and N2 data from RT-qPCR analysis of wastewater samples collected across the state of Minnesota from May to September. (**B**) Correlation and lag analysis of wastewater SARS-CoV-2 N1 levels with new daily clinical cases of COVID-19 for the state of Minnesota. (**C**) Correlation and lag analysis of wastewater SARS-CoV-2 N2 levels with new daily clinical cases of COVID-19 for the state of Minnesota.

To test the capacity of Melvin’s Index as a pathogen surveillance tool, we compared indexed values of SARS-CoV-2 genome copies/L from wastewater to new daily clinical COVID-19 cases. Statewide clinical data showed that the number of new COVID-19 cases peaked in late May, began to decrease until mid-July, and then steadily increased through August (Fig. 3A). Interestingly, statewide levels of target genes N1 and N2 showed a similar pattern to that of clinical cases of COVID-19 for the same period. However, the indexed N1 and N2 levels appeared to slightly precede new clinical cases (Fig. 3A). Trends similar to that of the statewide pattern were observed for five of the seven state regions (Fig. S3E,G).

### Melvin’s Index values of SARS-CoV-2 levels from wastewater predicted new clinical COVID-19 cases

Given the observed lead of indexed SARS-CoV-2 values over new clinical COVID-19 cases, we next tested for correlation between the two metrics while lagging index values were in successive one-day increments (Fig. 3B,C). Before lagging, statewide Melvin’s Index values showed low but significant correlation with new COVID-19 cases (*r* = 0.38 for both N1 and N2). Shifting the Melvin’s Index curve forward in time to maximize correlation showed that wastewater predicted confirmed new cases by 15d for N1 (*r*_107_ = 0.73, P < 0.001) and by 17d (*r*_107_ = 0.77, P < 0.001) for N2. When individual regions were considered, lag analysis showed less predictive power. Greatest agreement between N1 and N2 predictive windows was observed for the Central region (N1: 23d, *r*_98_ = 0.66, P < 0.001 and N2: 23d, *r*_98_ = 0.78, P < 0.001). Greatest correlation with confirmed new cases for both N1 and N2 Melvin’s Index values was observed for the North East region (Table S4).

## Discussion

In this study, we have demonstrated the effective application of wastewater surveillance to monitor disease prevalence and incidence across a large and variously populated geographic area. We created a normalized and standardized index, Melvin’s Index, that is robust to sewage system and population variation. The index provides a means of directly comparing pathogen prevalence between WWTFs and correlates with disease incidence. Further, as an example, we have shown that by using our method of indexing wastewater SARS-CoV-2 concentrations, WBE can presage changes in new COVID-19 clinical cases by as much as two weeks (15–17 days) at a statewide level.

Several sociological factors can influence correlation between Melvin’s Index and reported clinical COVID-19 cases. For example, wastewater SARS-CoV-2 RNA data from two regions of Minnesota (South East and South West) did not strongly correlate with clinical case trends (Table S4). This finding likely reflects circumstances unique to these two regions such as differences in COVID-19 testing, implementation of public health mitigation strategies, or other variables that are not present in other parts of the state. Of note, the South East region of Minnesota is a major manufacturing, agriculture product processing (with a migrant workforce), and healthcare hub for the state, all of which may have impacted the frequency of testing and geographical tagging of testing results. The South West region of Minnesota experienced large increase in COVID-19 cases prior to the commencement of our study which may have modified population behavior and testing frequency.

The extensive range of our sampling, in terms of both geographic and population size, provides further evidence that WBE is an effective and powerful public health implement for tracking disease incidence and prevalence across geographic areas. Additionally, this study serves as a step toward proof-of-concept studies that employ smaller, neighborhood-scaled sampling within larger population centers. In this study we have shown that wastewater monitoring can be implemented in populations as small as 500. Our study sets the stage for implementing a large network of surveillance sites for monitoring and prevention of future outbreaks across large geographic areas. Expansion of these successful strategies of WBE to include sequencing technologies for the surveillance of emerging SARS-CoV-2 variants should be a high priority. Additionally, WBE has great potential to track community-spread of seasonal and emerging respiratory or gastrointestinal pathogens to inform public health policy and control of disease spread.

## Methods

### Wastewater treatment plants

A major goal of this study was to determine the strategy for and effectiveness of large-scale WBE monitoring of disease prevalence across a large region such as a state in the U.S.A. To this end we studied WWTFs in the state of Minnesota, recruiting from the state’s major metropolitan area and six surrounding geographical areas designated as North West, North East, Central, South West, South Central, and South East (Fig. 1A). To recruit WWTFs, we solicited participation from the 57 members of the Minnesota Environmental Science and Economic Review Board (MESERB, meserb.org). An enrollment announcement was posted on MESERB’s news web page on April 17, 2020 (http://meserb.org/umd-seeks-facilities-to-participate-in-covid-19-wastewater-study). WWTF mangers were asked to submit a letter of intent to participate and commit to providing samples through August 31, 2020. Sizes of the population served by each participating WWTF plant, its designed average daily flow rate in millions of gallons per day (MGD), and size of its connected sewer system in kilometers were based on Omana et al.^22^. Minnesota population numbers were based on United States Census Bureau data (www.census.gov).

### Sample collection and viral inactivation

A total of 570 samples were collected (30 per site) from the 19 participating WWTFs. Wastewater was subsampled from 24-h composites of total plant influent collected by WWTF personnel. Subsamples consisted of 50–500 mL drawn from daily 24-h total plant intake composites. Collection frequency was once per week for the first month of participation in the study, and then once every two weeks until the end of the study. With the exception of the South East region, where only one WWTF participated, weekly monitoring at the regional level was maintained after the shift in sampling frequency by de-synchronizing submission from the WWTFs. The reduced sampling frequency was necessary for the distribution of limited laboratory staff and resources across the study period during COVID-19 stay at home restrictions in Minnesota.

Samples were shipped to the laboratory overnight on wet ice. A record of daily wastewater influent flow rate was included with each shipment. Upon receipt, the surfaces of sample tubes were wiped with 70% ethanol, bleached, and then placed into a 60 °C water bath for 2 h to pasteurize the samples. Following pasteurization, samples were stored at 4 °C until filtration. Storage times were typically 2 h or less.

### Viral precipitation and RNA extraction

Solid material was removed from the pasteurized samples by filtration through a 0.22 µm filter (MilliporeSigma SCGP00525). To precipitate viral particles, 35 mL of filtered wastewater was combined with 5 mL of 80% PEG-8000 and 0.3 M NaCl solution in a 50-mL OakRidge centrifuge tube, vortexed to mix thoroughly, and held on ice until centrifugation. Viral particles were pelleted by centrifugation at 12,000×*g* for 1.5 h, at 4 °C. To extract viral RNA, virus pellets were resuspended in Qiazol Lysis Reagent (Qiagen 79306) and incubated 5 min at room temperature. Aqueous and organic phases were separated by addition of chloroform, vortexing, and centrifugation at 12,000×*g* for 15 min at 4 °C. The aqueous phase was transferred to Qiagen miRNeasy mini spin columns (Qiagen 217004) for RNA purification using manufacturer protocol specifications for micro-RNA isolation. Purified RNA was stored at − 80 °C.

### Quantitative reverse transcriptase polymerase chain reaction (qRT-PCR) of wastewater viral load

The quantity of SARS-CoV-2 genomic RNA present in the extracted RNA samples was assayed using the CDC emergency use authorization quantitative reverse transcription polymerase chain reaction (qRT- PCR) protocol and Research Use Only (RUO) reagents. Two amplification targets within the nucleocapsid gene, N1 and N2, were reverse transcribed and amplified using premixed primer/hydrolysis probes (Integrated DNA Technologies 10006713) and Go-Taq Probe 1-Step qRT-PCR system 2X master mixes with dUTP (Promega A6102). Each 20-µL qRT-PCR contained 5 µL of extracted RNA, 1.5 µL primer/probe mix, 10 µL Go-Taq master mix, and 0.4 µL Go-Script RT mix. Reactions were performed using a Qiagen RotorGene Q thermal cycler with the following thermal profile: reverse transcription at 45 °C for 15 min; denature and polymerase activation at 95 °C for 2 min; followed by a 50-cycle two-step amplification profile of 95 °C for 5 s and 55 °C for 30 s. Reactions were performed in triplicate (3 measures) within each machine run and positive controls consisting of 200 copies of the amplification target (Integrated DNA Technologies 10006625) and no template controls were included in every run. No amplification was ever observed in the no template controls.

Quantification cycle (cycle threshold, C_T_) was determined using Qiagen Q-Rex software. Quantification of virus copy number was based on standard curves constructed from serial, tenfold dilutions of positive control plasmid containing the N target gene (Integrated DNA Technologies 10006625). Standard curves for N1 and N2 were estimated using quantile regression on the C_T_-values of the serial dilutions and were then applied to the median of the three measures (N1: Median C_T_ = 40.75–3.75(Log_10_ (Copy Number)); N2: Median C_T_ = 39.78–3.08 (Log_10_ (Copy Number)). Log_10_ (copies/L of wastewater) was calculated as: Log_10_ (genome copies/L) = Log_10_ [(Copies in RNA sample volume × (RNA elution volume/RNA volume in reaction)/35-mL sample] × 1000 mL/L].

To standardize qPCR data across the study period and WWTFs, we included reactions that contained primers and hydrolysis probes specific to the PMMoV. PMMoV qRT-PCR reactions contained forward primer 5’-GAGTGGTTTGACCTTAACGTTGA-3’, reverse primer 5’-TTGTCGGTTGCAATGCAAGT-3’ and probe 5’-VIC-CCTACCGAAGCAAATG-BHQ1 and were reverse transcribed and amplified as described above. Standard curves for PMMoV were estimated using quantile regression on the C_T_-values of serial dilutions of RNA extracted from Tabasco sauce (McIlhenny Co.)^23^ and were then applied to the median of the three measures (Median C_T_ = 40.75–3.57 log10 Copy Number).

### Calculation of Melvin’s Index of wastewater viral load

We developed a novel index of wastewater viral load, Melvin’s Index, that first normalizes the Log_10_ (genome copies/L) data obtained from the three viral targets and then standardizes based on the normalized PMMoV data. To calculate the index value, the mean of the two or three C_T_ measures was calculated for each WWTF, sample date, and target gene. When the mean of the three C_T_-values equaled 51 for a target gene, the data point was temporarily excluded from quantile normalization for that target gene. The quantity Log_10_ (genome copies/L) was calculated for the remaining data using target gene specific standard curves. Quantile normalization of the Log_10_ (genome copies/L) data was performed separately for the N1, N2, and PMMoV target genes. For each target gene, the distribution of Log_10_ (genome copies/L) was divided into 5 equal bins that each contained 20% of the data, with bin 1 containing the bottom 20 percent of the data, and bin 5 containing the top 20% of the data. The data falling into each bin was reassigned the value (*Q5*) of its respective bin (*Q5* = 1, 2, 3, 4, or 5). All C_T_ = 51 data points were then added back to the data set and assigned to bin 1. Following normalization, the data set consisted of *Q5*-normalized values of the target genes (*N1_Q5*, *N2_Q5*, and *PMMoV_Q5*) for each WWTF and sample date. N1 and N2 data were standardized to PMMoV by dividing their *Q5*-normalized values by that of PMMoV. The standardized data (*StdQ5*) were natural log transformed to produce linear scaling and multiplied by 10/*Ln(*5) to set the scale range from -10 to 10. We call the values produced by this last transformation “Melvin’s Index” (Fig. 3C).

Because Melvin’s Index values are centered on the median Melvin’s Index value for each target gene, the relative levels of virus can be compared over time within N1, N2, and PMMoV, but not between target genes or viruses. We were concerned that the loss of relative level might cause misinterpretation by a non-expert audience. For example, in Fig. 3C, the Melvin’s Index value of N2 rises above that of the highly abundant PMMoV virus. To prevent misinterpretation, we modified Melvin’s Index to maintain relative level relationships. To calculate the modified Melvin’s Index, the *StdQ5* is multiplied by the ratio of the median Log_10_ (genome copies/L) for the N gene of interest to that of PMMoV ($relStdQ5 =StdQ5\times \left(N \; median \; Log(Genome \; copies/L)/PMMoV \; median \; Log(Genome \; copies/L)\right)$. Finally, the *relStdQ5* is natural log-transformed and then scaled to set mMI(PMMoV) equal to 1.0 by adding the natural number (*e*) to Ln(RelStdQ5) and then dividing the sum by *e* $(Ln(RelStdQ5+e)/e)$ (Fig. 3C). R code that uses C_T_-values as input is available from https://github.com/glennesimmonsjr/dirtywatercooler.

#### Correlation of Melvin’s Index with testing data and its predictive value

We tested for correlation between values of Melvin’s Index and confirmed new cases during the study period by calculating the Pearson correlation coefficient between the two variables. To determine the potential predictive window provided by wastewater monitoring, defined as the time between observing an increase in SARS-CoV-2 RNA in wastewater and the appearance of confirmed new COVID-19 positive tests, we successively lagged Melvin’s Index values in 1-day increments and tested for correlation with confirmed new cases. The lag time that maximized correlation between Melvin’s Index and new confirmed cases was taken as the predictive window time in days. Correlation and lag analyses were performed on both statewide averaged Melvin’s Index and on regional Melvin’s Index values. Confirmed new case data was compiled from USAfacts.org^24^.

### Statistical analysis

All statistical analysis was performed using JMP Pro-15 (SAS Institute, Cary N.C.) or custom R scripts. R scripts are available from https://github.com/glennesimmonsjr/dirtywatercooler.

## Supplementary Information

Supplementary Information.
