# Supplementary material for: A novel wastewater-based epidemiology indexing method predicts SARS-CoV-2 disease prevalence across treatment facilities in metropolitan and regional populations
Source: Sci Rep. 2021 Nov 1;11:21368. doi: 10.1038/s41598-021-00853-y (PMC8560786; doi:10.1038/s41598-021-00853-y)
Supplement: Supplementary file 1 — Supplementary Information. [file 41598_2021_853_MOESM1_ESM.docx]

**A novel wastewater-based epidemiology indexing method predicts SARS-CoV-2 disease prevalence across treatment facilities in metropolitan and regional populations**

Richard G. Melvin^1^, Emily N. Hendrickson^1^, Nabiha Chaudhry^1^, Onimitein Georgewill^2^, Rebecca Freese^3^, Timothy W. Schacker^4^ and Glenn E. Simmons, Jr.^1,5*^

^1^Department of Biomedical Sciences, University of Minnesota Medical School – Duluth, MN

^2^National Summer Undergraduate Research Program, University of Maryland – College Park, MD

^3^Biostatistical Design and Analysis Center, Clinical and Translational Science Institute, University of Minnesota – Minneapolis, MN

^4^ Department of Medicine, University of Minnesota Medical School, Minneapolis, MN

^5^ Carcinogenesis and Chemoprevention Program, Masonic Cancer Center – Minneapolis, MN

*Corresponding Author: Glenn E. Simmons, Jr., Ph.D., Assistant Professor, University of Minnesota Medical School – Duluth, Department of Biomedical Sciences, [gsimmons@d.umn.edu](mailto:gsimmons@d.umn.edu) Phone: 218-726-8386

**Supplementary Methods and Results**

**SARS-CoV-2 limit of detection and recovery percent from wastewater.**

To determine the limit of detection (LoD) of SARS-CoV-2 genomic RNA in wastewater using our precipitation and RNA purification methods, we spiked known concentrations of heat-inactivated 2019-nCoV-2 (ATCC, VR-1986HK) into sterilized wastewater using a 10-fold dilution series (3.45 to 7.45 Log ( genome copies/ L)). Spiked viral particles from each dilution were precipitated and extracted as described above. Two RNA extractions were performed for each dilution and then pooled to produce sufficient RNA for the experiment. QRT-PCR reactions were performed using the described protocols for the N1 and N2 gene targets. Twenty reactions were performed for each dilution. The percent of the twenty qRT-PCR reactions that produced a positive result was plotted against the log scale concentration for each dilution, and a logistic regression approach was then applied. The logistic relationships were defined as

*% Positive PCR results =* 100/ (1 + e ^(-24.6 × (^ *^Log (genome copies/ L^* ^- 4.4))^) for N1 and

*% Positive PCR results =* 100/ (1 + e ^(-24.9 × (^ *^Log (genome copies/ L^* ^- 4.4))^ for N2. The LoD was calculated as the *Log(genome copies/ L)* for which *% Positive PCR results* = 95 as described in Burns & Valdivia, 2008 (REFERENCE). For N1 the LoD was 4.55 Log ( genome copies/ L) and for N2 the LoD was 4.56 Log ( genome copies/ L) (see equation below). A limitation of our analysis is the absence of a fine-scale dilution series to precisely elucidate the LoD. Future work will repeat the LoD experiment at a fine scale to produce a more accurate model.


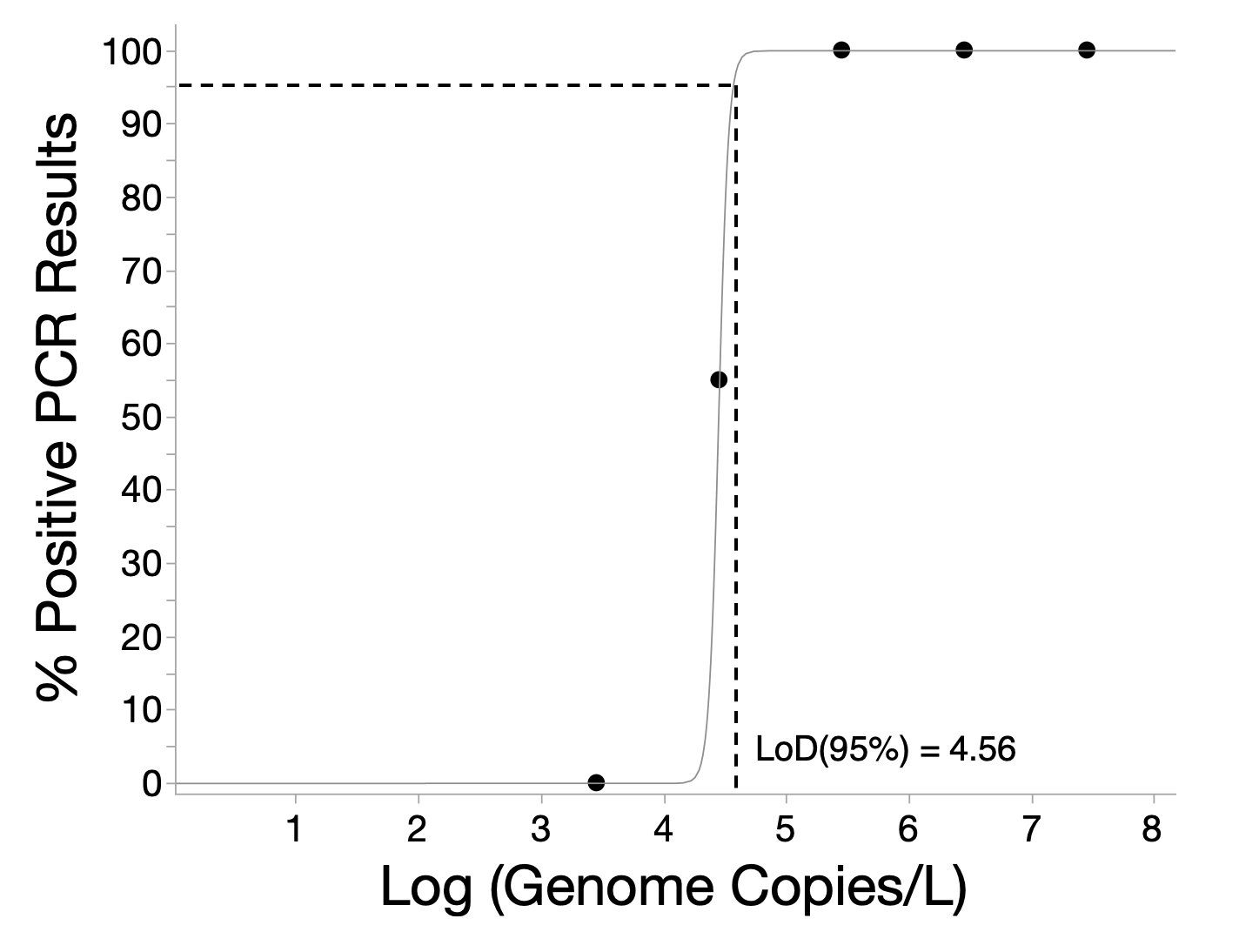


Logistic regression analysis of the concentration at 95% positive for the SARS-CoV-2 N2 target gene.


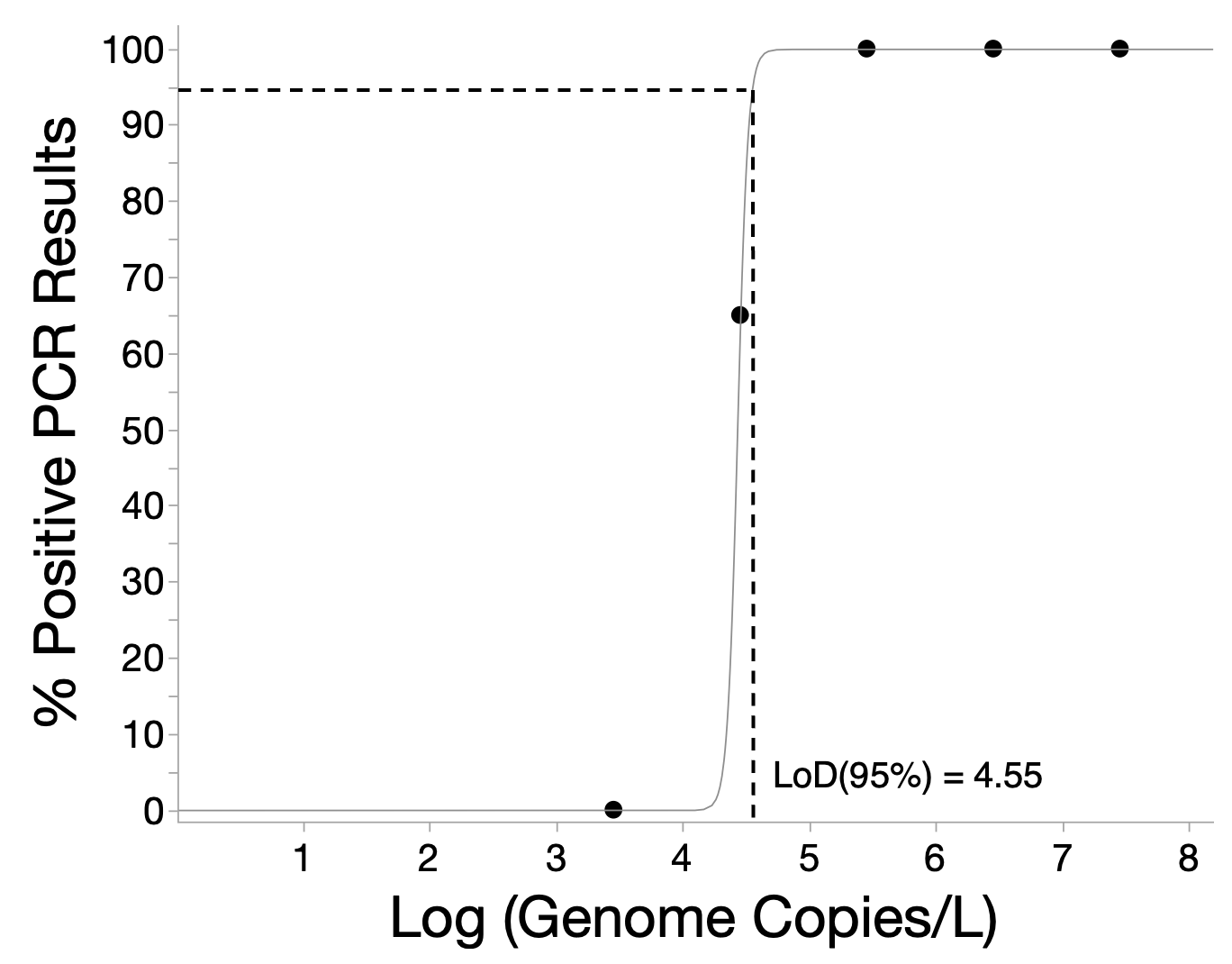


Logistic regression analysis of the concentration at 95 percent positive for the SARS-CoV-2 N1 target gene.

Recovery efficiency of SARS-CoV-2 RNA from wastewater samples using our methods was determined using the data provided by the LoD experiment. Recovery efficiency was calculated using the equation: % recovery = (total recovered genome copies per dilution/ total spiked genome copies per dilution) x 100%. Mean recovery efficiencies of target N1 were 18.3 ± 2.2 (mean ± standard deviation) and 27.1 ± 8.1 percent for 6.45 and 5.45 log (genome copies/ L), respectively. Mean recovery efficiencies of target N2 were 22.8 ± 8.4 and 24.5 ± 11.7 for 6.45 and 5.45 log (genome copies/ L), respectively. However, recovery efficiencies of the highest concentration, 7.45 log (genome copies/ mL), were 0.54% for N1 and 0.53% for N2. We hypothesize this effect is due to overloading the RNA purification column with viral nucleic acid. This would not influence the results from our study due to the low concentration of virus in wastewater. While our experimental design provides a generalized recovery efficiency, the presence of components in wastewater samples may produce an inhibitory effect on SARS-CoV-2 recovery and detection that our method does not account for. However, due to the analysis of PPMoV, in addition to SARS-CoV-2, any negative effects on PCR efficiency would presumably impact PCR amplification of both targets equally. This, to some degree, would be negated using Melvin’s Index to determine the relative amount of virus present.

**Tests for association of sewer system parameters on PMMoV standard C_T_-values.**

It was important to understand whether variation in system properties were associated with PMMoV levels because the global distribution of PMMoV C_T_ was used to calculate our SARS-CoV-2 level index (Melvin’s Index). Use of the global distribution assumes that the level of PMMoV in wastewater is fairly constant within and between populations and that there is a general relationship between PMMoV level and flow rate of the sewer system ^1–3^ . SARS-CoV-2 level, on the other hand, was expected to vary greatly within and between populations and be subject to the same associations with flow as is PMMoV.

We observed that WWTFs could be ranked according to their mean PMMoV C_T_-values (Figure S1D). This suggested a potential association with one or more site-specific variables. To better understand which site-specific variables influence the ranking of WWTFs by PMMoV C_T_, we tested whether flow rate, facility size (designed flow rate), population size, or the per capita measures of flow rate were associated with the ranking of WWTFs. When considering all of the participating WWTFs together, per capita flow rate was significantly associated with ranked PMMoV C_T_-values (Kendall’s τ = 0.15, P < 0.01, Table S2, Figure S1D). There was no significant association between PMMoV C_T_ ranking and flow rate, designed flow rate (facility size), size of the served population, or per capita measure of designed flow rate (Table 2).

Visual inspection of the per capita flow rate overlaid onto ranked PMMoV C_T_-values (Figure S1D) suggested that their association may be due to the concentration and dilution effects previously noted for two of the larger WWTFs. Metropolitan and WLSSD have strikingly different per capita flow rates (Figure S2B, S2D). The Metropolitan WWTF is a concentrated system that serves a population of 3,454,290 with a per capita flow rate of 0.55 ± 0.5 MGD/ 10k population (mean ± standard deviation) and PMMoV C_T_ of 25.17 ± 2.00. WLSSD is a dilute system that serves a population of 137,590 with a per capita flow rate of 2.23 ± 0.20 MGD/ 10k population (four times higher than that of Metropolitan) and PMMoV C_T_ of 28.44 ± 2.42. Significant association of the per capita flow rate with ranked PMMoV-C_T_ was lost when the WLSSD and Metropolitan WWTFs were removed from the analysis (Kendall’s τ = 0.08, P = 0.13), but not when either was removed individually (τ = 0.12, P < 0.05 and τ = 0.11, P < 0.05, respectively when Metropolitan or WLSSD were excluded) (Figure S2E, Table S2). Although rankable, PMMoV C_T_, did not differ significantly between WWTFs. This finding demonstrated that even large local differences in flow rate have a small effect on PMMoV C_T_ and supported both the use of PMMoV as a standard and the global PMMoV distribution in the calculation of Melvin’s index.

**References:**

1. Zhang, H., Bruns, M. A. & Logan, B. E. Biological hydrogen production by Clostridium acetobutylicum in an unsaturated flow reactor. *Water Res.* **40**, 728–734 (2006).

2. Kitajima, M., Rachmadi, A. T., Iker, B. C., Haramoto, E. & Gerba, C. P. Temporal variations in genotype distribution of human sapoviruses and Aichi virus 1 in wastewater in Southern Arizona, United States. *J. Appl. Microbiol.* **124**, 1324–1332 (2018).

3. Wu, F. *et al.* SARS-CoV-2 Titers in Wastewater Are Higher than Expected from Clinically Confirmed Cases. *mSystems* **5**, (2020).

**Figure Legends**

**Figure S1. Determining the influence of flow rates and other variables on Pepper Mild Mottle Virus detection in municipal wastewater samples.** A) Rank order of wastewater treatment facilities based on average daily flow rates. Error bars represent standard deviation of mean daily average flow rate for each site. B) Rank ordered wastewater treatment facilities based on daily average flow rates per capita. Error bars represent standard deviation of mean daily average flow rate for each site. C) Rank ordered wastewater treatment facilities based on the designed maximum daily flow rate per capita. D) The relationship between ranked order of wastewater treatment facilities based on PMMoV C_T_-values and the respective flow rates per capita. ANOVA showed that PMMoV C_T_ did not differ across WWTFs (F(_154,170_) = 0.60, P = 0.88).

E) The relationship between the ranked order of wastewater treatment facilities based on PMMoV C_T_-values and the respective flow rates per capita, excluding WLSSD and Metropolitan facilities. In D and E, Error bars represent standard deviation of mean daily average flow rate for each site (red) or mean PMMoV C_T_-values (gray). MGD, million gallons per day

**Figure S2. Normalizing and generation a novel index from RT-qPCR data obtained participating wastewater treatment facilities.** A) Comparison between new COVID-19 cases and the normalized, new COVID-19 cases per capita using two distinct geographic regions of Minnesota. B) Graphical depiction of the quantile normalization of SARS-CoV-2 N1, N2, and Pepper Mild Mottle Virus data. C) Step-by-step conversion of raw quantile data from N1, N2, and PPMoV gene targets to the normalized Melvin’s Index.

**Figure S3. Indexed SARS-CoV-2 levels in wastewater trends across the state show a similar pattern to new cases per capita with a temporal offset**. A, B, C, D, E, F, G) New daily clinical COVID-19 cases overlayed by indexed SARS-CoV-2 N1, N2, and the mean of N1 and N2 data from RT-qPCR analysis of wastewater collected different regions of the state of Minnesota.

**Tables**

**Table S1.** Characteristics of wastewater treatment plants (WWTP) included in the study and their regional population coverage.

| **WWTP** | **City Size Class^A^** | **Average Designed Flow^B^ (MGD)^C^** | **Sewer System Length^B^ (km)** | **Service Population^D^** | **Region** | **Region Population^D^** | **Region (%)** | **County** | **County Population^D^** | **County (%)** |
| --- | --- | --- | --- | --- | --- | --- | --- | --- | --- | --- |
| Thief River Falls | A | 3.0 | 70 | 8,570 | North West | 270,120 | 3.2 | Pennington | 14,120 | 60.7 |
| Moorhead | B | 4.5 | 310 | 46,340 |  |  | 17.1 | Clay | 64,220 | 72.2 |
| CIRSSD^E^ | A | 2.5 | 30 | 6,490 | North East | 465,040 | 1.4 | St. Louis | 199,070 | 3.3 |
| WLSSD^F^ | C | 48.4 | 1250 | 137,590 |  |  | 29.6 | St. Louis | 199,070 | 58.0 |
|  |  |  |  |  |  |  |  | Carlton | 35,871 | 61.8 |
| NKASD^G^ | A | 3.0 | 60 | 6,840 |  |  | 1.5 | Koochiching | 12,299 | 55.6 |
| Little Falls | A | 2.4 | 100 | 8,700 | Central | 652,410 | 1.3 | Morrison | 33,386 | 26.1 |
| Fergus Falls | B | 2.8 | 150 | 13,350 |  |  | 2.0 | Otter Tail | 58,746 | 22.7 |
| Willmar | B | 7.5 | 180 | 19,610 |  |  | 3.0 | Kandiyohi | 43,199 | 45.4 |
| Cambridge | A | 1.9 | 90 | 8,220 | Metropolitan | 3,313,061 | 0.2 | Isanti | 40,596 | 20.2 |
| Metropolitan^H^ | C | 314.0 | 2510 | 1,931,620 |  |  | 58.3 | Anoka |  |  |
|  |  |  |  |  |  |  |  | Dakota |  |  |
|  |  |  |  |  |  |  |  | Hennepin |  |  |
|  |  |  |  |  |  |  |  | Ramsey |  |  |
|  |  |  |  |  |  |  |  | Washington |  |  |
| New Prague | A | 1.8 | 70 | 7,510 |  |  | 0.2 | Scott | 149,010 | 5.0 |
| Northfield | B | 5.2 | 130 | 20,700 |  |  | 4.1 | Rice | 67,000 | 31.0 |
| Worthington | B | 4.0 | 100 | 13,000 | South West | 158,270 | 8.2 | Nobles | 21,630 | 60.1 |
| Marshall | B | 4.5 | 130 | 13,680 |  |  | 8.6 | Lyon | 25,470 | 53.7 |
| Lafayette | A | 0.1 | 5 | 500 | South Central | 269,420 | 0.2 | Nicollet | 34,300 | 1.4 |
| LeSueur | A | 1.8 | 50 | 4,060 |  |  | 1.5 | LeSueur | 28,900 | 14.0 |
| Hutchinson | B | 5.4 | 120 | 13,980 |  |  | 5.2 | McLeod | 35,890 | 38.9 |
| Mankato | B | 11.2 | 323 | 39,310 |  |  | 14.6 | Blue Earth | 67,650 | 58.1 |
| Rochester | C | 23.8 | 790 | 118,900 | South East | 511,310 | 23.3 | Olmsted | 158,300 | 75.1 |

| ^A^ City classification by population size. A: < 10,000; B: 10,000 to 100,000; and C: > 100,000. |
| --- |
| ^B^ Omana, *et* al. 2020. Future wastewater infrastructure needs and capital costs. Minnesota Pollution Control Agency, Document number Irwq-wwtp-1sy20. |
| ^C^ MGD = Millions of Gallons per Day. |
| ^D^ United States Census Bureau. B01001 SEX BY AGE, 2019 American Community Survey 5-Year Estimates. U.S. Census Bureau, American Community Survey Office. Web. 10 December 2020. http://www.census.gov/. |
| ^E^ **Central Iron Range Sanitary Sewer District** includes the **St. Louis County** communities of Chisholm (4,814), Buhl (969), Kinney (144), and Great Scott Township (561). |
| ^F^ **Western Lake Superior Sanitary District** includes the **St. Louis** **County** communities of Duluth (86,210), Hermantown (9,630), Proctor (3,030), Rice Lake (4,140), Canosia (2,160), Duluth township (1,940), Grand Lake township (2,780), Lakewood township (2,190), Midway township (1,400), and Solway township (1,940); and the **Carlton** county communities of Cloquet (12,120), Carlton (1,020), Scanlon (960), Thomson (160), Wrenshall (400), Thomson township (5,000), Silver Brook township(600), and Twin Lakes township (1,910). |
| ^G^ **North Koochiching Area Sanitary District** includes the **Koochiching County** communities of International Falls (5,695), Rainier (548), and Jackfish Bay (600). |
| ^H^ **Met Council Metropolitan WWTP** includes the **Anoka** county communities of Andover (31,690), Anoka (17,140), Blaine (65,500), Centerville (3,790), Circle Pines (5,010), Columbia Heights (19,500), Columbus (3,890), Coon Rapids (61,480), Fridley (27,210), Hilltop (770), Lexington (2,050), Ramsey (24,810), and Spring Lake Park (6,510); the **Washington** county communities of Birchwood (870), Forest Lake (19,130), Hugo (13,880), Lake Elmo (8,070), Landfall (730), Lino Lakes (21,120), Mahtomedi (8,030), Newport (3,440), Oakdale (27,540), St. Paul Park (5,440), Willernie (500), and Woodbury (69,240); the **Hennepin** county communities of Brooklyn Center (30,100), Brooklyn Park (81,000), Champlin (24,010), Corcoran (5,510), Crystal (22,150), Dayton (5,010), Edina (47,940), Golden Valley (20,850), Hopkins (17,590), Maple Grove (70,000), Medicine Lake (370), Medina (4,890), Minneapolis (427,960), New Hope (20,340), Osseo (2,500), Plymouth (78,400), Richfield (35,380), Robbinsdale (14,760), Rogers (12,540), St Anthony (9,080), and St. Louis Park (47,220); the **Ramsey** county cities of Arden Hills (9,550), Falcon Heights (5,320), Gem Lake (500), Lauderdale (2,500), Little Canada (9,800), Maplewood (39,770), Mounds View (13,010), New Brighton (21,460), North Oaks (5,020), North St. Paul (11,460), Roseville (34,600), Shoreview (26,500), St. Paul (294,870), Vadnais Heights (12,300), White Bear Lake (24,070), and White Bear Township (11,000); and the **Dakota** county cities Inver Grove Heights (35,230), Lilydale (600), Mendota (200), Mendota Heights (11,070), South St. Paul (20,160), and West St. Paul (19,540). |

***Table S2.*** *Tests of association between system factors and ranking of PMMoV C_T_-values.*

| **Y** | **X** | **Slope** | **Intercept** | **Kendall τ** | ***n*** | **P > \|τ\|** |
| --- | --- | --- | --- | --- | --- | --- |
| PMMoV C_T_-value | Flow Rate (MGD) | 0.01 | 27.51 | 0.02 | 19 | 0.69 |
| PMMoV C_T_-value | Designed Flow Rate (MGD) | -0.11 | 28.13 | − 0.03 | 19 | 0.55 |
| PMMoV C_T_-value | Served Population | 1.59E-6 | 27.51 | – 0.01 | 19 | 0.84 |
| PMMoV C_T_-value | Flow Rate per 10,000 population (MGD/10k) | 2.15 | 24.0 | 0.15 | 19 | < 0.01** |
|  |  | ^A^1.98 | 24.28 | 0.08 | 17 | 0.13 |
| PMM0V C_T_-value | Designed Flow Rate per 10,000 population (MGD/10k) | 0.55 | 26.23 | 0.03 | 19 | 0.50 |

^A^ WLSSD and Metropolitan WWTFs excluded.

**Table S3.** Principal components analysis on correlations. Percent of variation contained by each principal component and the loading for each variable.

|  |  | **PC1** | **PC2** | **PC3** | **PC4** |
| --- | --- | --- | --- | --- | --- |
| **All WWTFs** | |  |  |  |  |
| **Percent of variation** | | 56.13 | 18.05 | 15.26 | 10.27 |
| **Loading** | Population | 0.97 | 0.20 | 0.08 | 0.03 |
|  | Designed flow rate | 0.97 | 0.23 | 0.10 | 0.02 |
|  | Flow rate | 0.96 | 0.24 | 0.10 | 0.00 |
|  | C_T_ | − 0.18 | 0.63 | -0.75 | 0.06 |
|  | Flow rate/10k | − 0.48 | 0.57 | 0.38 | -0.54 |
|  | Designed Flow/10k | − 0.54 | 0.46 | 0.45 | 0.57 |
|  |  |  |  |  |  |
| **Exclude WLSSD and Metropolitan** | | |  |  |  |
| **Percent of variation** | | 49.59 | 20.91 | 16.04 | 12.56 |
| **Loading** | Population | 0.99 | 0.01 | 0.00 | 0.04 |
|  | Designed flow rate | 0.97 | 0.16 | − 0.16 | 0.16 |
|  | Flow rate | 0.96 | 0.21 | − 0.07 | − 0.11 |
|  | C_T_ | − 0.01 | 0.40 | 0.91 | 0.05 |
|  | Flow rate/10k | − 0.19 | 0.77 | − 0.22 | − 0.56 |
|  | Designed Flow/10k | − 0.32 | 0.65 | − 0.26 | − 0.63 |

**Table S4.** Lag analysis comparing Melvin’s Index to new COVID-19 cases/ 10k population.

| **Region** | **Probe** | **Lag (days)** | **Correlation (*r*)** | | ***n*** | **P** |
| --- | --- | --- | --- | --- | --- | --- |
| Minnesota | N1 | 15 | 0.73 | 107 | | < 0.001 |
|  | N2 | 17 | 0.77 | 107 | | < 0.001 |
|  | Mean N1, N2 | 17 | 0.68 | 107 | | < 0.001 |
| North West | N1 | 25 | − 0.14 | 101 | | 0.17 |
|  | N2 | 18 | 0.57 | 107 | | < 0.001 |
|  | Mean N1, N2 | 22 | 0.30 | 107 | | < 0.01 |
| North East | N1 | 18 | 0.84 | 107 | | < 0.001 |
|  | N2 | 4 | 0.84 | 107 | | < 0.001 |
|  | Mean N1, N2 | 11 | 0.86 | 107 | | < 0.001 |
| Central | N1 | 23 | 0.66 | 98 | | < 0.001 |
|  | N2 | 23 | 0.78 | 98 | | < 0.001 |
|  | Mean N1, N2 | 23 | 0.76 | 98 | | < 0.001 |
| Metropolitan | N1 | 5 | 0.40 | 107 | | < 0.001 |
|  | N2 | 11 | 0.83 | 107 | | < 0.001 |
|  | Mean N1, N2 | 7 | 0.77 | 107 | | < 0.001 |
| South West | N1 | 0 | − 0.4 | 106 | | 0.65 |
|  | N2 | 21 | − 0.19 | 106 | | 0.055 |
|  | Mean N1, N2 | 0 | − 0.14 | 106 | | 0.14 |
| South Central | N1 | 4 | 0.35 | 100 | | < 0.001 |
|  | N2 | 1 | 0.61 | 100 | | < 0.001 |
|  | Mean N1, N2 | 4 | 0.53 | 100 | | < 0.001 |
| South East | N1 | 25 | 0.38 | 105 | | < 0.001 |
|  | N2 | 25 | − 0.30 | 105 | | < 0.01 |
|  | Mean N1, N2 | 25 | − 0.04 | 105 | | 0.67 |

**
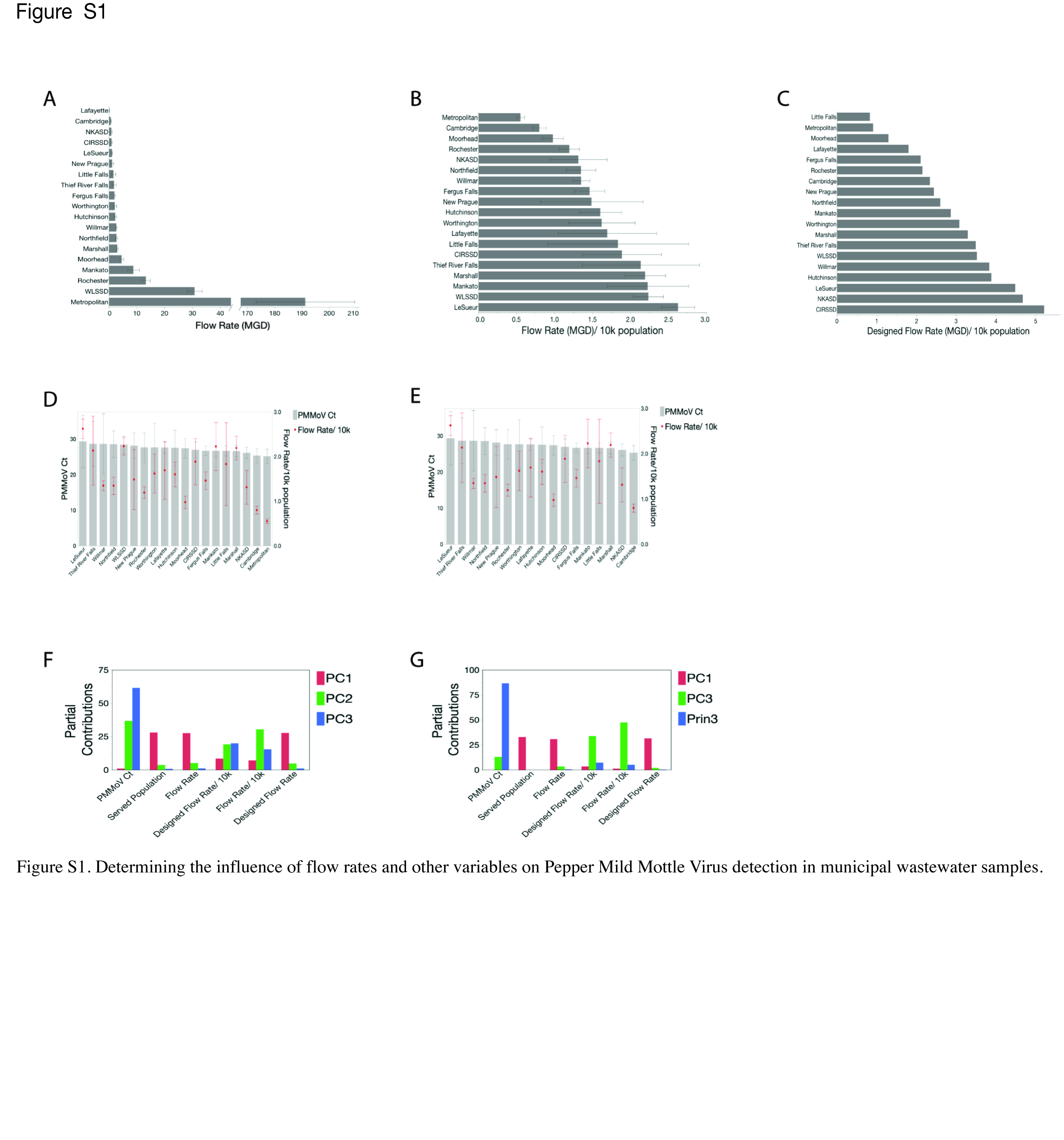
**

**
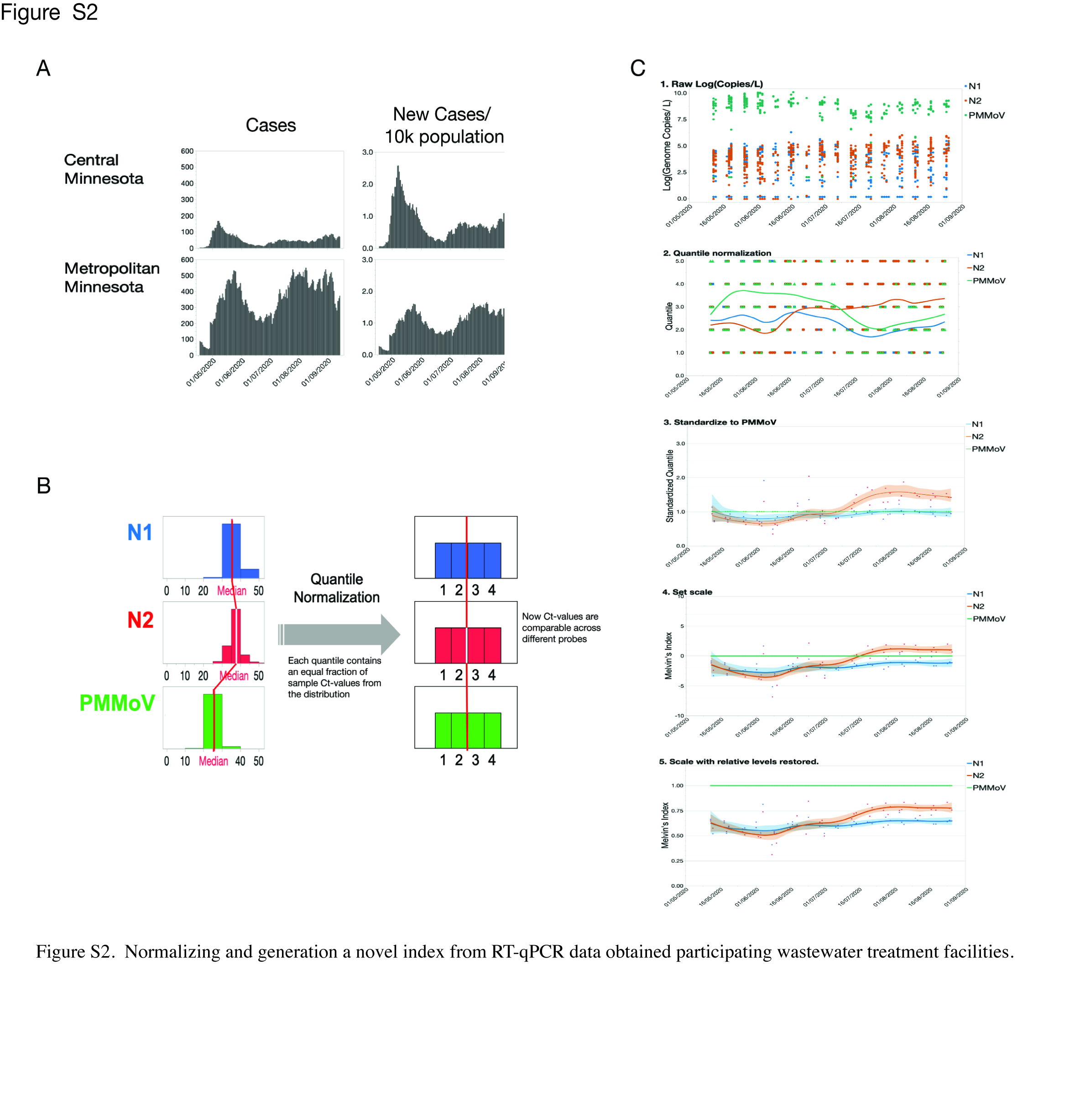
**

**
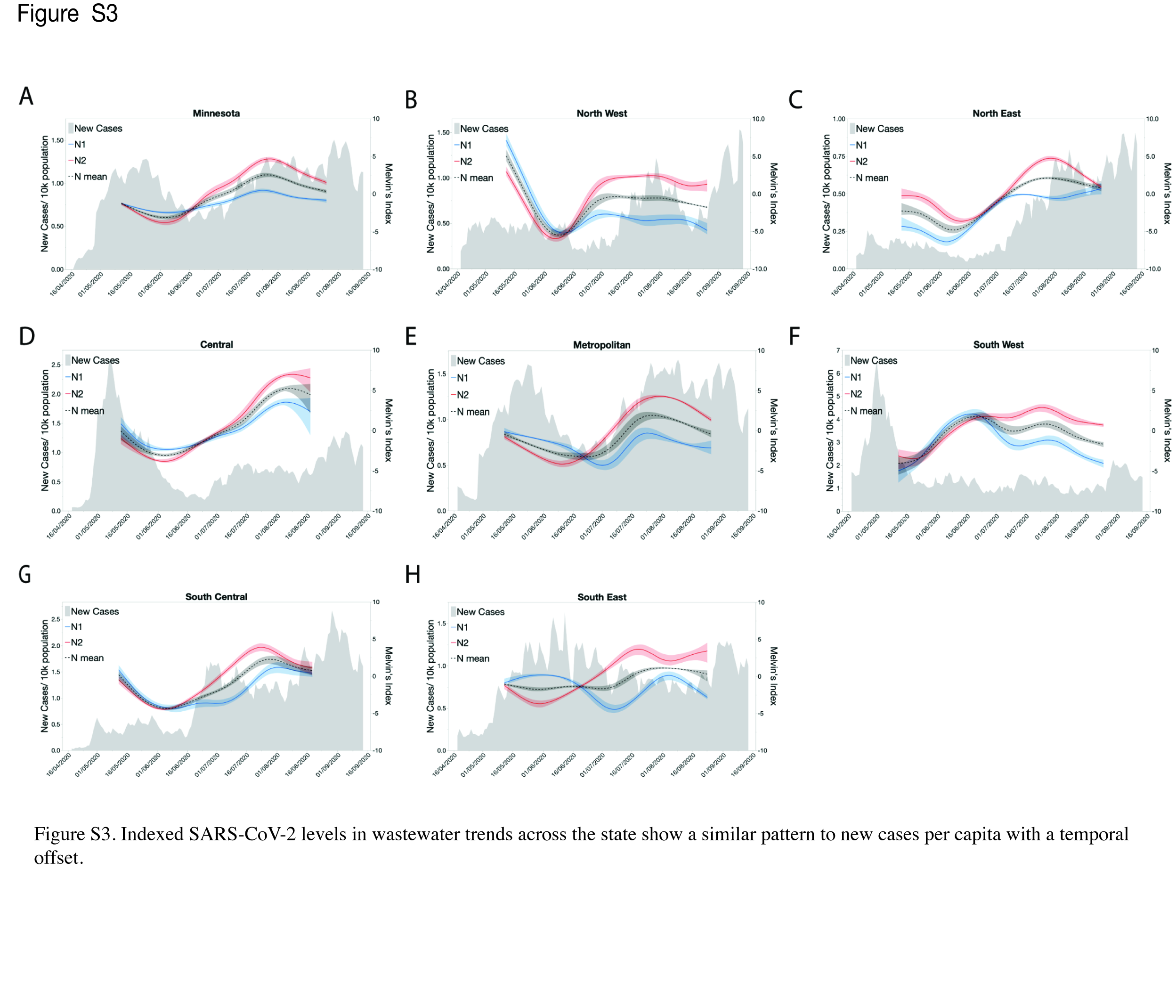
**
